# 肺癌胸腔镜肺叶切除、胸腔镜肺段切除与开胸肺叶切除术后对肺功能影响的研究

**DOI:** 10.3779/j.issn.1009-3419.2016.10.15

**Published:** 2016-10-20

**Authors:** 艳娇 张, 禹舜 高

**Affiliations:** 100021 北京，北京协和医学院中国医学科学院肿瘤医院胸外科 Department of Toracic Surgical Oncology, Cancer Institute and Hospital, Chinese Academy of Medical Sciences and Peking Union Medical College, Beijing 100021, China

**Keywords:** 肺肿瘤, 肺功能, 微创手术, Lung neoplasms, Pulmonary function, Minimally invasive surgery

## Abstract

**背景与目的:**

肺癌是世界范围内发病率和死亡率最高的恶性肿瘤之一，手术仍然是早期非小细胞肺癌的首选治疗方法。本研究的目的是探讨术后极早期肺功能的恢复情况，并比较肺癌胸腔镜肺叶切除、胸腔镜肺段切除与开胸肺叶切除术后对肺功能影响。

**方法:**

选取中国医学科学院肿瘤医院胸外科2015年9月-2016年2月间手术的肺癌患者，按术式不同分为胸腔镜肺段切除术组、胸腔镜肺叶切除术组、开胸肺叶切除术组，分别于术前、术后第3天和术后3个月检查测试肺功能。统计分析采用SPSS 20.0版本，应用单因素方差分析，比较组间差异。

**结果:**

① 在术后第3天，对比胸腔镜肺叶切除术、胸腔镜肺段切除术、开胸肺叶切除术，三组患者的肺功能，用力肺活量（forced vital capacity, FVC）、FVC占预计值的百分比（FVC%）、一秒用力呼气容积（forced expiratory volume in one second, FEV1）、FEV1占预计值的百分比（FEV1%）、最大呼气流速峰值（peak expiratory?ow, PEF）、每分钟最大通气量（maximal voluntary ventilation, MVV）、肺一氧化碳弥散因子（transfer factor for carbon monoxide of lung, TLCO）、TLCO占预计值的百分比（TLCO%），组间差异具有统计学意义（P值分别为0.033、0.042、0.029、0.045、0.039、0.021、0.018、0.024）。②在术后3个月，对比胸腔镜肺叶切除术、胸腔镜肺段切除术、开胸肺叶切除术，三组患者的肺功能，组间比较发现FVC、FVC%、FEV1、FEV1%、PEF、MVV、TLCO、TLCO%差异显著（P值分别为0.019、0.024、0.044、0.021、0.037、0.029、0.045、0.017）。

**结论:**

肺癌胸腔镜肺叶切除、胸腔镜肺段切除与开胸肺叶切除术后在术后极早期（术后第3天）与术后3个月，肺功能的恢复情况均为胸腔镜肺段切除优于胸腔镜肺叶切除，胸腔镜肺叶切除优于开胸肺叶切除。

## Abstract (en)

**Background and objective:**

Lung cancer is a malignancy with high morbidity and mortality rates worldwide. Surgery is the preferred treatment for non-small cell lung cancer. This study aims to investigate the effects of video-assisted thoracoscopic surgery (VATS) lobectomy, VATS segmentectomy, and open thoracotomy on pulmonary function in the early postoperative stage and compare the difference among three groups.

**Methods:**

Pulmonary function data of patients were collected from September 2015 to February 2016 in Department of Thoracic Surgical Oncology, Cancer Hospital Chinese Academy of Medical Sciences. The patients were categorized according to operation methods into three groups, namely, VATS segmentectomy, VATS lobectomy, and open thoracotomy groups. Pulmonary function was assessed 1 day before the surgery, 3 days after the surgery, and 3 months after the surgery. Statistical analysis was performed with SPSS 20.0 through single-factor analysis of variance.

**Results:**

Pulmonary function 3 days after the surgery was compared among the three groups. There was a significant difference in forced vital capacity (FVC), FVC%, forced expiratory volume in one second (FEV1), FEV1%, peak expiratory flow (PEF), maximal voluntary ventilation (MVV), transfer factor for carbon monoxide of lung (TLCO) and TLCO% (*P* values were as follows: 0.033, 0.042, 0.029, 0.045, 0.039, 0.021, 0.018, 0.024). The comparison of pulmonary function of 3 groups at the time of 3 months after operation showed that there was a significant difference among three groups in FVC, FVC%, FEV1, FEV1%, PEF, MVV, TLCO, TLCO% (*P* values were as follows: 0.019, 0.024, 0.044, 0.021, 0.037, 0.029, 0.045, 0.017).

**Conclusion:**

No matter in the early stage after surgery or at the time of 3 months after surgery, the patients' pulmonary function in VATS segmentectomy is better than the data of VATS lobectomy group, and the pulmonary function data of VATS lobectomy patients recovered better than the open thoracotomy group.

肺癌是世界范围内发病率和死亡率最高的恶性肿瘤之一，手术仍然是早期非小细胞肺癌的首选治疗方法，微创化是外科手术的发展趋势，电视胸腔镜手术（video-assisted thoracoscopic surgery, VATS）是实现微创化的重要途径。胸腔镜微创肺叶切除术与肺段切除术具有切口美观、恢复快、创伤小以及对肺功能影响小的优点^[1]^。但是在术后极早期（术后3天）对肺功能的影响有多少，国内外均未有较全面的报道，本研究旨在探讨术后极早期肺功能的恢复情况，为临床评价手术安全性提供重要依据，并比较开胸肺叶切除、胸腔镜肺叶切除术、胸腔镜肺段切除术术后肺功能的恢复情况。

## 1 资料与方法

### 1.1 临床资料

收集中国医学科学院肿瘤医院胸外科2015年9月-2016年2月间手术的肺癌患者，分别于术前、术后第三天和术后三个月检查测试肺功能。按照术式不同分为胸腔镜肺段切除术组，胸腔镜肺叶切除术组，开胸肺叶切除术组。胸腔镜肺段切除术组患者60例，中位年龄56岁，男性30例，女性30例。胸腔镜肺叶切除术组有56例，中位年龄54岁，男性27例，女性29例。开胸肺叶切除术组42例，中位年龄60岁，男性22例，女性20例。行开胸肺叶切除术的原因有：肿瘤直径＞5 cm；中央型肺癌，与大血管关系密切。入组标准：①肺部病变，经胸片、电子计算机断层扫描（computed tomography, CT）检查有手术指征；②年龄＞18岁，心、肺、肝、肾等主要器官功能无手术禁忌证；③非小细胞肺癌，临床分期为Ⅲa期以前；④术前未行放疗，无严重的胸腔粘连。排除标准：①有活动性结核病史；②广泛胸膜粘连；③不适合单肺通气；④既往有过开胸手术史；⑤未在术后第3天及术后3个月行肺功能检查者。三组患者术前资料组间比较肺功能差异均无统计学意义（*P*＞0.05，表 1）。

**1:** 三组患者术前资料比较 Comparison of preoperative pulmonary function among three groups

Variable	VATS segmentectomy group	VATS lobectomy group	Open thoractomy	*F*	*P*
FVC (L)	2.98±0.95	2.95±0.38	2.75±0.69	2.522	0.082
FVC%	85.97±12.28	85.86±11.22	82.71±15.26	0.086	0.914
FEV1 (L)	2.58±0.83	2.42±0.65	2.38±0.61	3.528	0.068
FEV1%	90.43±13.53	86.89±12.61	84.36±18.05	1.483	0.234
FEV1/FVC	85.22±6.75	80.29±9.16	80.25±9.45	2.275	0.104
PEF (L/S)	5.08±1.35	4.29±1.68	4.89±2.37	0.885	0.436
MVV (L/min)	82.53±28.17	79.87±24.92	75.53±19.41	4.132	0.089
TLCO (mmol/min/kPa)	7.94±1.98	8.09±1.35	7.43±1.48	4.753	0.098
TLCO (mmol/min/kPa)%	93.95±16.21	95.15±16.23	86.44±13.79	5.038	0.086
FVC: forced vital capacity; FVC%: FVC/pred; FEV1: forced expiratory volume in one second; FEV1%: FEV1/pred; PEF: peak expiratory flow; MVV: maximal voluntary ventilation; TLCO: transfer factor for carbon monoxide of lung; TLCO%: TLCO/pred; VATS: video-assisted thoracoscopic surgery.					

### 1.2 方法

使用德国耶格公司生产的Jaeger Msdiffusion肺功能仪进行检查，术前肺功能为必测，术后测定术后第3天和/或术后3个月时的肺功能。手术方法：术前准备和手术体位，三组均相同，均采取双腔气管插管，健侧单肺通气，患者侧卧位。VATS组切口选择为三孔法或单孔法。三孔法：胸腔镜孔一般选择腋前线第7或8肋间；主操作孔选择腋前线第4或第5肋间，切口长度约2 cm，以正对肺门结构为原则；副操作孔位于听诊三角处，切口长度1.5 cm。单孔法：腋中线第4肋间，切口长度约2 cm，器械、镜头均经此孔进行操作。开胸组常规选取侧后外侧切口，逐层进胸后，术中尽量避免对正常肺组织的牵拉和挤压，常规行肺叶切除及纵隔肺门淋巴结清扫。观察指标包括：用力肺活量（forced vital capacity, FVC）、FVC占预计值的百分比（FVC%）、一秒用力呼气容积（forced expiratory volume in one second, FEV1）、FEV1占预计值的百分比（FEV1%）、FEV1/FVC、最大呼气流速峰值（peak expiratory flow, PEF）、每分钟最大通气量（maximal voluntary ventilation, MVV）、肺一氧化碳弥散因子（transfer factor for carbon monoxide of lung, TLCO）、TLCO占预计值的百分比（TLCO%）。

### 1.3 统计学方法

统计分析采用SPSS 20.0版本，应用方差分析单因素*ANOVA*检验比较组间差异，*P*＜0.05为差异有统计学意义。

## 2 结果

在术后第3天，对比胸腔镜肺叶切除术、胸腔镜肺段切除术、开胸肺叶切除术，三组患者的肺功能（FVC、FVC%、FEV1、FEV1%、PEF、MVV、TLCO、TLCO%）组间差异具有统计学意义（*P*值分别为0.033、0.042、0.029、0.045、0.039、0.021、0.018、0.024）（表 2）。在术后3个月，对比胸腔镜肺叶切除术、胸腔镜肺段切除术、开胸肺叶切除术，三组患者的肺功能组间比较发现FVC、FVC%、FEV1、FEV1%、PEF、MVV、TLCO、TLCO%差异显著（*P*值分别为0.019、0.024、0.044、0.021、0.037、0.029、0.045、0.017）（表 3）。

**2:** 术后第3天三组患者肺功能比较 Comparison of pulmonary function at the time of 3 days after surgery among 3 groups

Variable	VATS segmentectomy group	VATS lobectomy group	Open thoractomy	*F*	*P*
FVC (L)	1.66±0.59	1.35±0.38	1.09±0.27	3.587	0.033
FVC%	44.39±10.29	36.80±10.74	30.00±4.12	3.036	0.042
FEV1 (L)	1.55±0.49	1.26±0.35	1.00±0.18	3.965	0.029
FEV1%	48.35±12.09	39.19±12.04	35.54±6.27	2.937	0.045
FEV1/FVC	92.39±10.12	90.77±10.26	94.21±9.45	1.375	0.299
PEF (L/S)	4.93±1.22	3.72±0.83	2.86±0.75	3.976	0.039
MVV (L/min)	50.29±20.23	43.24±11.29	38.84±6.16	4.124	0.021
TLCO (mmol/min/kPa)	4.26±0.56	3.32±0.48	2.44±0.16	4.267	0.018
TLCO (mmol/min/kPa)%	51.48±10.56	40.80±10.74	30.10±4.10	4.093	0.024

**3:** 术后3个月三组患者肺功能比较 Comparison of pulmonary function at the time of 3 months after surgery among 3 groups

Variable	VATS segmentectomy group	VATS lobectomy group	Open thoracotomy group	*F*	*P*
FVC (L)	2.98±1.02	2.52±0.47	1.96±0.58	5.767	0.019
FVC%	81.19±14.58	73.27±12.07	62.21±15.38	5.065	0.024
FEV1 (L)	2.36±0.74	1.99±0.32	1.61±0.42	3.256	0.044
FEV1%	86.74±13.29	77.11±11.62	69.94±14.07	5.372	0.021
FEV1/FVC	82.23±10.13	81.39±7.98	75.05±9.89	1.293	0.282
PEF (L/S)	6.17±1.39	5.29±1.56	4.28±1.49	3.834	0.037
MVV (L/min)	82.78±29.58	62.34±20.15	49.79±18.46	4.578	0.029
TLCO (mmol/min/kPa)	7.29±2.08	6.23±1.69	4.58±1.24	3.128	0.045
TLCO (mmol/min/kPa)%	77.01±24.24	63.74±20.83	47.20±16.89	5.822	0.017

## 3 讨论

胸腔镜微创手术在保证根治性的前提下，与开胸手术相比，具有术后疼痛轻、并发症发生率低、术后恢复快的优点^[2]^，而对于病变小于2 cm的Ia期肺癌患者而言，胸腔镜肺段切除与胸腔镜肺叶切除术相比，又能进一步减少对肺功能的损失，提高术后患者生活质量。美国国立综合癌症网络（National Comprehensive Cancer Network, NCCN）指南也推荐胸腔镜肺叶切除术为可切除的肺癌的标准术式。胸腔镜手术能够大大减少术后发生呼吸衰竭等并发症的几率，但是仍有极少数患者因高龄，有慢支、肺气肿、弥漫性肺大泡合并重度通气功能障碍，加之术前呼吸道准备不够，手术时间过长，健侧肺过度通气，术后拔除气管插管过早等原因，容易出现呼吸衰竭。研究胸腔镜手术对肺功能的影响，对于进一步减少术后并发症发生率、确保手术安全性有着重要意义。车国卫等^[3]^的研究认为，FEV1和PEF实测值于术后第7天在VATS组（1.64±0.21, 310.58±30.13）显著高于开胸组（1.34±0.11, 270.18±25.67）（*P*＜0.05），而术后第30天两组患者FEV1（1.68±0.32, 1.65±0.09）和PEF（330.33±26.19, 307.73±32.14）实测值无统计学差异（*P*＞0.05）。然而，目前尚未有研究报道开胸肺叶切除术、胸腔镜肺叶切除术和胸腔镜肺段切除术后第3天早期肺功能的变化。

影响肺功能的因素诸多，如吸烟、高龄、性别、身高、体质量、被动吸烟情况、严重胸廓畸形、患有某些肺部疾病。术后早期患者往往因为疼痛，而限制胸廓进行呼吸运动，因此笔者认为术后疼痛可能为影响肺功能的重要因素，而术后3天以内也是围手术期与呼吸相关并发症发生率较高的时期，因此测定术后3天时不同术式患者肺功能的情况具有重要意义。术后3个月，患者疼痛较术后围手术期大大减轻，胸壁肌肉愈合良好，各方面情况趋于稳定，身体素质有所恢复，因此比较术后3个月肺功能的恢复情况，可以评价不同术式对患者生活质量的影响。

本研究在术后第3天，测试不同术式患者的肺功能，FVC、FVC%、FEV1、FEV1%、PEF、MVV、TLCO、TLCO%各组间差异均有统计学意义（*P*＜0.05）。魏学强等^[4]^通过对比31例行VATS肺叶切除患者与30例行开胸手术患者的术后第3天的肺功能，结果显示：VC（1.42±0.32 *vs* 1.17±0.45, *t*=2.41, *P*＜0.05）、FVC（1.36±0.36 *vs* 1.08±0.36, *t*=2.95, *P*＜0.05）、FEV1（1.06±0.37 *vs* 0.85±0.25, *t*=2.72, *P*＜0.05）、MVV（47.35±17.67 *vs* 30.49±9.37, *t*=4.82, *P*＜0.05）差异显著。李迎新等^[5]^测定开胸肺叶切除与胸腔镜肺叶切除术后第7天患者的肺功能，经过分析，VATS组患者术后第7天FEV1、MVV、PEF与传统开胸组FEV1、MVV、PEF相比较差异有统计学意义（*P*＜0.05）。传统开胸手术切断胸壁肌肉、肋骨、肋间肌肉及肋间神经；胸骨撑开器过度牵拉胸壁引起的损伤，造成术后早期患者疼痛，术后限制型呼吸障碍；同时术中操作时压迫肺脏，使其挫伤较严重，引起肺间质及呼吸膜（肺泡膜）水肿，肺泡表面活性物质减少或破坏。胸腔镜手术创伤变小，缩短了开、关胸时间，减少了对肋骨及肋间神经的损伤，使术后恢复时间缩短。因而开胸组肺功能损伤较为严重。英国爱丁堡皇家医院Oparka等^[6]^的一篇文章中指出胸腔镜肺叶切除组与开胸肺叶切除组相比，术后肺炎的发病率显著降低（4.3% *vs* 21.7%, *P*＜0.05），因此对肺功能的影响更小。胸腔镜肺段切除术与胸腔镜肺叶切除术相比，差别在于肺容积的损失不同，胸腔镜肺段切除术能够在保证根治性的前提下，保留更多的正常的肺组织，术后肺功能的恢复优于胸腔镜肺叶切除术组。

术后3个月，胸腔镜肺段切除术组、胸腔镜肺叶切除术组及开胸肺叶切除术组比较，除FEV1/FVC外，肺功能各项指标差异均有统计学差异（*P*值分别为0.019、0.024、0.044、0.021、0.037、0.029、0.045、0.017），胸腔镜肺段切除术组肺功能的恢复情况优于胸腔镜肺叶切除术组，胸腔镜肺叶切除组优于开胸肺叶切除术组。在肺叶切除术后，随着同侧剩余肺组织的膨胀代偿，肺功能水平逐渐提高^[7]^，肺组织的膨胀作用从一定程度上可以弥补部分肺叶切除术后对肺功能的损失，胸廓肋间改变，胸膜机化填充，纵隔偏移及膈肌的抬高都起到一定的作用。虽然人体自身为适应术后身体环境的改变做出了一些改变，但是这些长期的慢性的改变仍不足以弥补手术方式的不同引起肺功能的损失，开胸肺叶切除术后，受损的肋间肌肉为瘢痕组织替代，这将是永久性的不可逆的改变，因此开胸肺叶切除术后呼吸肌的损伤是不可忽视的影响术后远期肺功能恢复的一个因素。特别是病变所在肺叶切除后，同侧剩余肺叶膨胀会逐步取代切除肺叶的空间，这种取代，会造成剩余肺叶的支气管角度的变形，使气道变狭窄，增加了气道阻力^[8]^，而肺段切除组几乎不受支气管角度变化的影响，这种支气管角度的改变可能是造成两组MVV有差异的原因。韩国首尔国立大学盆唐医院的Kim等^[9]^研究300例非小细胞肺癌患者术后3个月、术后1年的肺功能变化，其中行胸腔镜肺叶切除术227例，胸腔镜肺段切除术73例，认为胸腔镜肺段切除术在肺功能恢复方面优于胸腔镜肺叶切除组，能更好地保留患者的肺功能，差异有统计学意义（*P*＜0.001）。我们的研究中胸腔镜肺段切除组患者肺功能在术后3个月恢复优于胸腔镜肺叶切除术组，且各项指标差异具有统计学意义（*P*＜0.05）。胸腔镜肺叶切除术后，尽管余肺的膨胀可以起到一定的代偿作用，仍不足以弥补肺组织本身的损伤对肺功能造成的改变。

胸腔镜微创手术与传统开胸手术相比，对于患者术后肺功能的恢复具有明显的优势，而胸腔镜肺段切除术与胸腔镜肺叶切除术相比，也能更好地保留肺功能。进一步证实需要大规模大样本量的随机分组对照研究。
